# Advancing preoperative planning technology in total joint arthroplasty with a real-time machine learning calculator: 1-year mortality risk in a value-based care era

**DOI:** 10.1186/s42836-026-00428-0

**Published:** 2026-09-09

**Authors:** Alexander H. King, Kole Joachim, Christopher D. Hamad, Casey Abernethy, Aaron Cheng, Timothy Ko, Brandon Gettleman, Adrian Lin, Amanda Perrotta, Sumin Jeong, Ezekiel Dingle, Joshua M. Wiener, Alexandra Stavrakis, Alexander B. Christ

**Affiliations:** 1 Department of Orthopaedic Surgery, University of California, Los Angeles, CA 90095 USA; 2 David Geffen School of Medicine at the University of California, Los Angeles, CA 90095 USA; 3 Department of Orthopaedic Surgery, Greater Los Angeles VA Medical Center, Los Angeles, CA 90073 USA

**Keywords:** Total joint arthroplasty, Machine learning, Mortality risk prediction, Preoperative risk stratification, Value-based care

## Abstract

**Background:**

Technological innovation in total joint arthroplasty (TJA) has largely focused on intraoperative precision through robotics, navigation, and implant design, while preoperative decision-making remains comparatively underdeveloped. Accurate estimation of patient-specific risk is central to surgical indications, yet existing tools provide limited resolution for consequential outcomes such as 1-year mortality.

**Methods:**

A machine learning model was developed using the TriNetX Research Network. Patients undergoing total knee (TKA) or hip (THA) arthroplasty with verifiable 1-year follow-up were included in the analysis. An XGBoost model incorporating 43 preoperative variables was trained using nested cross-validation with isotonic calibration. Model performance was assessed using discrimination, calibration, and decision curve analysis, and the results were used to derive thresholds for a web calculator based on machine-learning-derived inputs. Feature contributions were assessed using SHapley Additive exPlanations (SHAP).

**Results:**

The observed 1-year mortality rate was 0.574% (*n* = 1,344). On internal validation, the model demonstrated an Area Under the Receiver Operating Characteristic (AUROC) of 0.761 (fold-level range: 0.744–0.787, standard deviation [SD]: 0.016), a Brier score of 0.006, and an average precision of 0.037. Calibration remained stable across risk deciles, with mean absolute error below 0.15% for D1–D9 and modest overprediction confined to D10 (+ 0.16%). Mortality increased monotonically across stratified risk groups: the highest quintile had a 1.488% mortality rate (2.6-fold above baseline), the highest decile had 2.50% (4.4-fold), and the top 5% had 3.57% (6.2-fold). Feature importance analysis identified age as the dominant predictor (mean |SHAP| = 0.407), followed by male sex (0.218), serum creatinine (0.162), hemoglobin (0.153), and BMI (0.145). Threshold-based risk stratification yielded two clinically relevant operating points: a sensitivity-anchored threshold of 0.235% captured 90.1% of all deaths, while a Youden-optimal threshold of 0.767% achieved 83.9% specificity.

**Conclusions:**

One-year mortality following primary THA and TKA can be estimated with discriminative accuracy using routinely available preoperative variables, with predictive importance attributable primarily to age and markers of physiologic reserve. Supported by these findings and future external validations, machine learning-derived mortality models are amenable to prospective clinical deployment via web-based interfaces, enabling individualized preoperative risk quantification at the point of care.

**Supplementary Information:**

The online version contains supplementary material available at https://doi.org/10.1186/s42836-026-00428-0.

## Introduction

Technology has reshaped how total joint arthroplasty (TJA) is performed. Robotic assistance, computer navigation, and patient-specific instrumentation have advanced intraoperative precision over the past decade. However, preoperative decision-making has not kept pace. Who should undergo surgery, when, and after what optimization remain questions largely answered with categorical instruments developed before modern data infrastructure existed. This gap matters because elective TJA volume continues to climb, with projected increases of 71% for total hip arthroplasty (THA) and 85% for total knee arthroplasty (TKA) by 2030 [1]. At this scale, even low-incidence adverse outcomes carry substantial population-level burden.

Among these outcomes, mortality remains the most consequential. Reported 1-year mortality rates after primary TJA range from approximately 0.5% to 1.6%, with higher risk observed in older patients and those with greater medical complexity [2]. The 1-year time horizon is particularly relevant in elective arthroplasty, as patients who die within this period are unlikely to realize the durable improvements in pain and function that justify surgery. Although several risk factors are well established, including advanced age, cardiovascular disease, renal dysfunction, malnutrition, and frailty, translating these factors into an individualized risk estimate remains challenging [2, 3]. Risk does not distribute uniformly within comorbidity categories, and the interactions between age, laboratory values, and physiologic reserve are often nonlinear. As a result, applying population-level associations to individual patients at the point of care remains imprecise, highlighting the need for more individualized approaches to preoperative risk estimation.

Existing risk stratification tools reflect these limitations. Instruments such as the ASA Physical Status classification, Charlson Comorbidity Index (CCI), and modified Frailty Index (mFI) assign similar risk to all patients within a given category, limiting their ability to capture variation in clinical risk [4, 5]. More comprehensive calculators such as the ACS-NSQIP Surgical Risk Calculator often show poor calibration in TJA populations, with predicted event rates that diverge from observed outcomes [6]. The shared limitations are straightforward. Continuous physiologic variation is collapsed into ordinal categories, and predictors are assumed to act in additive or linear combinations. Machine learning (ML) methods offer a potential solution by modeling nonlinear interactions across a large set of variables without requiring a prespecified functional form. Gradient-boosted tree algorithms such as XGBoost are particularly well suited to this task given their ability to model nonlinear interactions without requiring a prespecified functional form. Performance of these models depends on the data and clinical context in which they are applied. In prior orthopaedic studies, reported Area Under the Receiver Operating Characteristic (AUROC) values have ranged from 0.72 to 0.94 for short-term outcomes including 30-day mortality, readmission, and composite complications [7–10]. Prior work has shown that individualized estimation of 1-year mortality after TJA is feasible, with registry-based tools such as JointCalc providing web-based risk calculators. However, these models rely on a limited set of variables and are not designed to incorporate more granular physiologic data or integrate into real-time clinical workflows available in modern EMR-based care [11]. Extending these approaches into clinician-facing, point-of-care tools represents the next step in preoperative planning. A model built on routinely available preoperative data that produces individualized risk estimates in real time could better inform patient selection, optimization, and surgical timing.

Therefore, the primary objective of this study was to develop an ML-based preoperative risk calculator for 1-year all-cause mortality after primary THA and TKA. Secondary aims included assessing clinical utility using decision curve analysis, identifying the top preoperative predictors using SHAP-based feature importance, and releasing the model as a free, real-time clinical calculator to support preoperative planning.

## Methods

### Study design and data source

We conducted a retrospective cohort study using the TriNetX Research Network (Cambridge, MA, USA), a federated platform aggregating de-identified electronic health records from U.S. health systems. The Research Network includes over 160 million patients across 111 healthcare organizations. All data in TriNetX are de-identified under the Health Insurance Portability and Accountability Act (HIPAA) Privacy Rule (45 CFR §164.514[b][1]) using expert determination methods and minimum cell sizes (≥ 10). This study does not constitute human subjects research per 45 CFR §46.102 and is exempt from institutional review board oversight. The TriNetX Research Network was queried in October of 2025 to generate the line-level data used for cohort construction for this analysis.

### Cohort identification, follow-up ascertainment, and cohort restriction

Patients undergoing primary THA or TKA were identified using International Classification of Diseases, Tenth Revision, Procedure Coding System (ICD-10-PCS) codes. The index date was defined as the date of the qualifying arthroplasty procedure. Because TriNetX captures data from patients with ongoing interactions within participating healthcare organizations, patients without continued follow-up may appear as censored survivors even if they died outside the network. To address this censoring bias, the primary analysis cohort was restricted to patients who met at least one of the following criteria: (1) confirmed death within 365 days of the index surgery; or (2) at least 365 days of observable post-surgical follow-up, defined as at least one clinical encounter recorded in the TriNetX database at least 365 days after the index surgery date. This restriction excluded 75,277 patients (24.3% of the full cohort of 309,529) whose 1-year survival status could not be verified.

### Outcome definition

The primary outcome was 1-year all-cause mortality, defined as death from any cause within 12 calendar months after the index arthroplasty. Date of death was available only by month and year per TriNetX de-identification standards; the day of death was conservatively imputed as the first day of the reported month.

### Feature selection and engineering

Candidate predictors were drawn from preoperative clinical data available on or before the index surgery date, including demographic variables (age, sex, body mass index [BMI], procedure type), preoperative laboratory values (creatinine, hemoglobin, white blood cell count, glucose, albumin, platelet count, hemoglobin A1c, international normalized ratio), vital signs (systolic and diastolic blood pressure), comorbidity flags (16 conditions), ASA physical status class, and healthcare utilization in the prior 12 months (inpatient hospitalizations and emergency department visits). ASA physical status class was not directly recorded in TriNetX and was approximated using a hierarchical ICD-10-based algorithm assigning class IV for organ failure, dialysis, or recent myocardial infarction or stroke, class III for heart disease, COPD, chronic kidney disease, obstructive sleep apnea, or insulin-dependent diabetes, class II for hypertension, non-insulin-dependent diabetes, or thyroid disease, and class I for none of the above. This derived variable was used as a continuous predictor in the model and as a baseline comparator for discrimination analysis. A total of 43 features were included in the final model. Missing laboratory values were imputed using population-level medians computed from the training cohort. Missingness in TriNetX laboratory data is non-random, as test ordering reflects clinical indication and care patterns. Accordingly, binary missingness indicator variables were included as model features for all eight laboratory predictors to allow the model to learn differential risk associated with absent preoperative testing. Model-based multiple imputation was not used out of concern that iterative imputation within a cross-validated pipeline could introduce leakage whereby imputed values informed by the full-sample outcome distribution would risk artificially optimistic performance estimates. Preoperative utilization variables (prior hospitalizations, prior emergency department visits) were winsorized at the 99th percentile of the training distribution to reduce the influence of extreme outliers.

### Model development

An XGBoost gradient-boosted tree classifier was trained on the primary analysis cohort to predict 1-year all-cause mortality. Hyperparameters were set to 500 estimators, a learning rate of 0.05, a maximum tree depth of 5, a minimum child weight of 50, a subsample fraction of 0.8, and a column subsample fraction of 0.7. Class imbalance was addressed using isotonic regression for probability calibration. A single model was trained on the combined THA and TKA cohort to maximize training sample size, with procedure type encoded as binary indicator features. To assess the appropriateness of this approach, a post-hoc subgroup analysis was conducted in which SHAP values were computed separately for THA and TKA patients and applied to a random sample of 5,000 patients per subgroup, and predictor rankings were compared across procedure types.

### Internal validation and calibration

Model performance was assessed using a nested, stratified fivefold cross-validation framework to generate out-of-fold predicted probabilities for all patients, ensuring separation between training and evaluation data. Probability calibration was performed with isotonic regression within the cross-validation framework to preserve independence between model fitting and calibration. All performance metrics were computed from out-of-fold predictions and therefore represent internally validated estimates. Discrimination was quantified by AUROC. Calibration was assessed using decile-based calibration tables comparing predicted and observed event rates. The Brier score and area under the precision-recall curve (AUPRC) were reported as supplementary metrics. Subgroup performance was evaluated separately for THA and TKA.

### Threshold derivation

Three complementary approaches were applied to the out-of-fold (OOF) calibrated probabilities to identify relevant risk thresholds. First, a decision curve analysis (DCA)-based threshold was derived by identifying the predicted probability that maximized net benefit across a pre-specified risk range of 0.5% to 10%, selected based on the observed event rate (0.574%) and clinically actionable risk levels. Second, a Youden index threshold, defined as the predicted probability that maximizes the sum of sensitivity and specificity, was calculated as the mean of fold-specific thresholds across cross-validation folds, with the standard deviation (SD) reported as a measure of stability. Third, a sensitivity-anchored threshold was defined as the minimum predicted probability that achieved ≥ 90% sensitivity on the full OOF prediction set to prioritize high-risk case identification.

### Feature importance

SHAP (SHapley Additive exPlanations) values for the final full-cohort XGBoost model were computed using the TreeExplainer algorithm on a random sample of 5,000 patients. Mean absolute SHAP values were used to rank features by their average contribution to model predictions.

### Statistical analysis

Continuous variables are reported as medians (interquartile ranges [IQR]) and categorical variables are reported as counts and percentages. Between-group comparisons (THA versus TKA) used the Mann–Whitney U test (two-sided) for continuous variables and the Pearson chi-square with Yates continuity correction for categorical variables. *p* < 0.05 was considered statistically significant and associated 95%-confidence intervals (95%-CI) are reported. All analyses were performed in Python 3. The final calibrated model was deployed as a web-based clinical calculator using Streamlit, enabling real-time risk estimation based on user-entered preoperative variables. The deployed tool uses the final model trained on the full cohort and preserves the same preprocessing and calibration pipeline as described above. It is available at the following hyperlink. (https://tjamortalitycalculator.streamlit.app/).

## Results

### Cohort characteristics

The primary analysis cohort comprised 234,252 patients undergoing primary TJA who met the adequate follow-up criterion (Table 1). Procedures included 140,204 TKAs (59.9%) and 94,048 THAs (40.1%). Surgeries were performed between 2000 and 2025, with 88.9% of the cohort (*n* = 208,351) undergoing surgery in 2015 or later. The remaining 11.1% (*n* = 25,898) were performed prior to 2015, with 1.8% (*n* = 4,277) prior to 2010. The median age at surgery was 66 years (IQR 60–73), and 58.3% of patients were male. THA patients were significantly younger than TKA patients (median 65.0 vs 67.0 years; *p* < 0.001) and less likely to be male (54.7% vs 60.8%; *p* < 0.001). The median BMI was 30.4 kg/m^2^ (IQR 26.6–35.0). THA patients had a lower BMI than TKA patients (median 29.0 vs 31.4 kg/m^2^; *p* < 0.001). The most prevalent comorbidities were hypertension (50.8%), obstructive sleep apnea (14.6%), anemia (14.1%), thyroid disease (13.8%), heart disease (10.1%), anticoagulation therapy (12.1%), and malignancy (7.4%). Diabetes mellitus was present in 8.8%, and chronic obstructive pulmonary disease (COPD) in 4.7%. In the 12 months preceding surgery, 14.4% had at least one inpatient hospitalization and 11.2% had at least one emergency department visit. The 1-year all-cause mortality rate in the primary cohort was 0.574% (*n* = 1,344). Mortality was significantly higher among THA patients (0.647%; *n* = 608) than among TKA patients (0.525%; *n* = 736; *p* < 0.001). Among the 1,344 patients who died within 365 days of surgery, the median time from surgery to death was 188 days (IQR 87–278 days; 6.2 months, IQR 2.9–9.2). A total of 131 deaths (9.7%) occurred within 30 days of surgery and 343 (25.5%) within 90 days, while 1,001 patients (74.5%) died between 91 and 365 days postoperatively. Median time to death did not differ significantly between THA (192 days, IQR 90–283) and TKA patients (183 days, IQR 86–272; *p* = 0.1), with a similar proportion of deaths occurring within 90 days in each group (25.2% vs 25.8%).

**Table 1:** Baseline characteristics of the primary analysis cohort, stratified by procedure type

Variable	Overall (*n* = 234,252)	THA (*n* = 94,048)	TKA (*n* = 140,204)	*p*-value
**Demographics**				
Age at surgery, years [median (IQR)]^a^	66 (60, 73)	65 (58, 72)	67 (61, 73)	< 0.001
Male sex, No. (%)	136,664 (58.3)	51,432 (54.7)	85,232 (60.8)	< 0.001
BMI, kg/m^2^ [median (IQR)]	30.4 (26.6, 35.0)	29.0 (25.5, 33.4)	31.4 (27.5, 36.0)	< 0.001
**Comorbidities**				
Hypertension, No. (%)	119,048 (50.8)	45,163 (48.0)	73,885 (52.7)	< 0.001
Diabetes mellitus, No. (%)	20,575 (8.8)	6,879 (7.3)	13,696 (9.8)	< 0.001
Heart disease,d No. (%)	23,765 (10.1)	9,312 (9.9)	14,453 (10.3)	0.001
Anemia, No. (%)	32,921 (14.1)	13,895 (14.8)	19,026 (13.6)	< 0.001
Active malignancy, No. (%)	17,432 (7.4)	7,378 (7.8)	10,054 (7.2)	< 0.001
Obstructive sleep apnea, No. (%)	34,130 (14.6)	11,799 (12.5)	22,331 (15.9)	< 0.001
COPD, No. (%)	10,973 (4.7)	4,765 (5.1)	6,208 (4.4)	< 0.001
Chronic kidney disease, No. (%)	14,239 (6.1)	5,339 (5.7)	8,900 (6.3)	< 0.001
Anticoagulation therapy, No. (%)	28,372 (12.1)	10,934 (11.6)	17,438 (12.4)	< 0.001
Thyroid disease, No. (%)	32,248 (13.8)	12,111 (12.9)	20,137 (14.4)	< 0.001
Comorbidity count [median (IQR)]	1 (0, 2)	1 (0, 2)	1 (0, 2)	< 0.001
**Preoperative laboratory values**				
Creatinine, mg/dL [median (IQR)]^b^	0.9 (0.7, 1.0)	0.9 (0.7, 1.0)	0.9 (0.7, 1.0)	< 0.001
Hemoglobin, g/dL [median (IQR)]^b^	13.5 (12.5, 14.5)	13.5 (12.3, 14.5)	13.5 (12.5, 14.5)	< 0.001
WBC, × 10^3^/µL [median (IQR)]^b^	6.8 (5.7, 8.3)	6.9 (5.7, 8.5)	6.8 (5.6, 8.1)	< 0.001
Glucose, mg/dL [median (IQR)]^b^	101 (91, 119)	100 (91, 120)	101 (91, 118)	0.126
Albumin, g/dL [median (IQR)]^b^	4.0 (4.0, 4.1)	4.0 (4.0, 4.1)	4.0 (4.0, 4.1)	< 0.001
Platelets, × 10^3^/µL [median (IQR)]^b^	244 (205, 288)	245 (206, 289)	243 (204, 287)	< 0.001
**Preoperative vitals**				
Systolic BP, mmHg [median (IQR)]^b^	128 (113, 142)	127 (112, 142)	129 (114, 143)	< 0.001
Diastolic BP, mmHg [median (IQR)]^b^	73 (63, 82)	73 (63, 82)	73 (64, 82)	< 0.001
**Healthcare utilization, prior 12 months**				
Any inpatient hospitalization, No. (%)	33,769 (14.4)	13,437 (14.3)	20,332 (14.5)	0.149
Any emergency department visit, No. (%)	26,260 (11.2)	10,185 (10.8)	16,075 (11.5)	< 0.001
**Primary outcome**				
1-year all-cause mortality, No. (%)	1,344 (0.57)	608 (0.65)	736 (0.52)	< 0.001

*Abbreviations*: BMI, body mass index; BP, blood pressure; CAD, coronary artery disease; COPD, chronic obstructive pulmonary disease; HF, heart failure; IQR, interquartile range; THA, total hip arthroplasty; TKA, total knee arthroplasty; WBC, white blood cell count

^a^ Continuous variables reported as median (IQR); *p*-values from Mann–Whitney U test (two-sided)

^b^ Missing laboratory values imputed to cohort median in model training. Missing rates: creatinine 16.8%, hemoglobin 12.5%, WBC 37.3%, glucose 13.6%, albumin 4.2%, platelets 27.5%, systolic and diastolic BP 11.7%

### Model performance

**Fig. 1:**
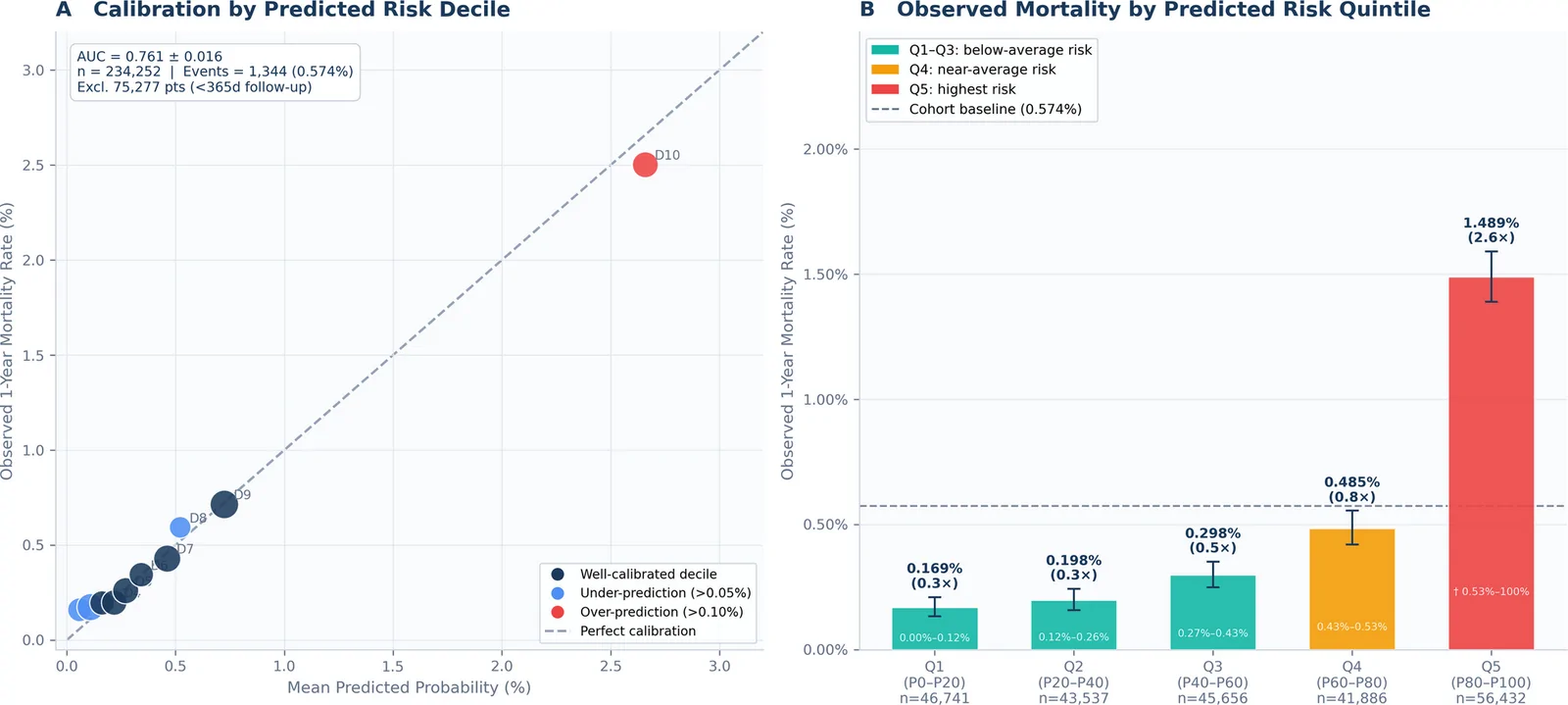
Model calibration and risk stratification for 1-year all-cause mortality after primary total joint arthroplasty. **A** Decile calibration plot comparing mean predicted and observed 1-year mortality rates across ten equal-sized bins of predicted risk. Error bars represent 95% confidence intervals for observed event rates. The dashed diagonal line indicates perfect calibration. **B** Observed 1-year mortality rate stratified by predicted risk quintile (Q1–Q5), where quintiles are defined by predicted-probability bins. Fold enrichment relative to the cohort baseline rate (0.574%) is displayed above each bar. The horizontal dashed line indicates the cohort baseline mortality rate

The XGBoost model achieved an internally validated AUROC of 0.761 (fold-level range: 0.744–0.787; standard deviation [SD]: 0.016). THA and TKA subgroup AUROCs were 0.778 and 0.746, respectively, representing a discrimination improvement of + 0.113 AUC over comorbidity count alone (AUC 0.648). The receiver operating characteristic curves for the XGBoost model and both baseline comparators are presented in Supplemental Fig. S1. The Brier score was 0.006, and the average precision was 0.037. Given that AUPRC is mathematically bounded by the event rate, this value reflects the constraint imposed by a 0.574% outcome prevalence rather than model performance. Calibration by predicted risk decile is shown in Fig. 1A. Across deciles D1 through D9, mean predicted probabilities closely tracked observed event rates, with mean absolute calibration error below 0.15 percentage points in each decile. Deciles (D4, D5, D7, D9) were well-calibrated, showing both predicted and observed rates clustering near the cohort baseline. Deciles D1, D2, D3, D6 were slightly underpredicted. D10 showed modest overprediction (+ 0.16 percentage points, mean predicted 2.66% vs observed 2.50%), attributable to a small number of patients with extreme feature combinations. These patients represented 0.35% of the cohort and did not materially affect aggregate calibration. At the procedure level, mean predicted and observed mortality were 0.648% vs 0.646% for THA and 0.524% vs 0.525% for TKA, confirming calibration was consistent across procedure types.

### Risk stratification and feature importance

**Fig. 2:**
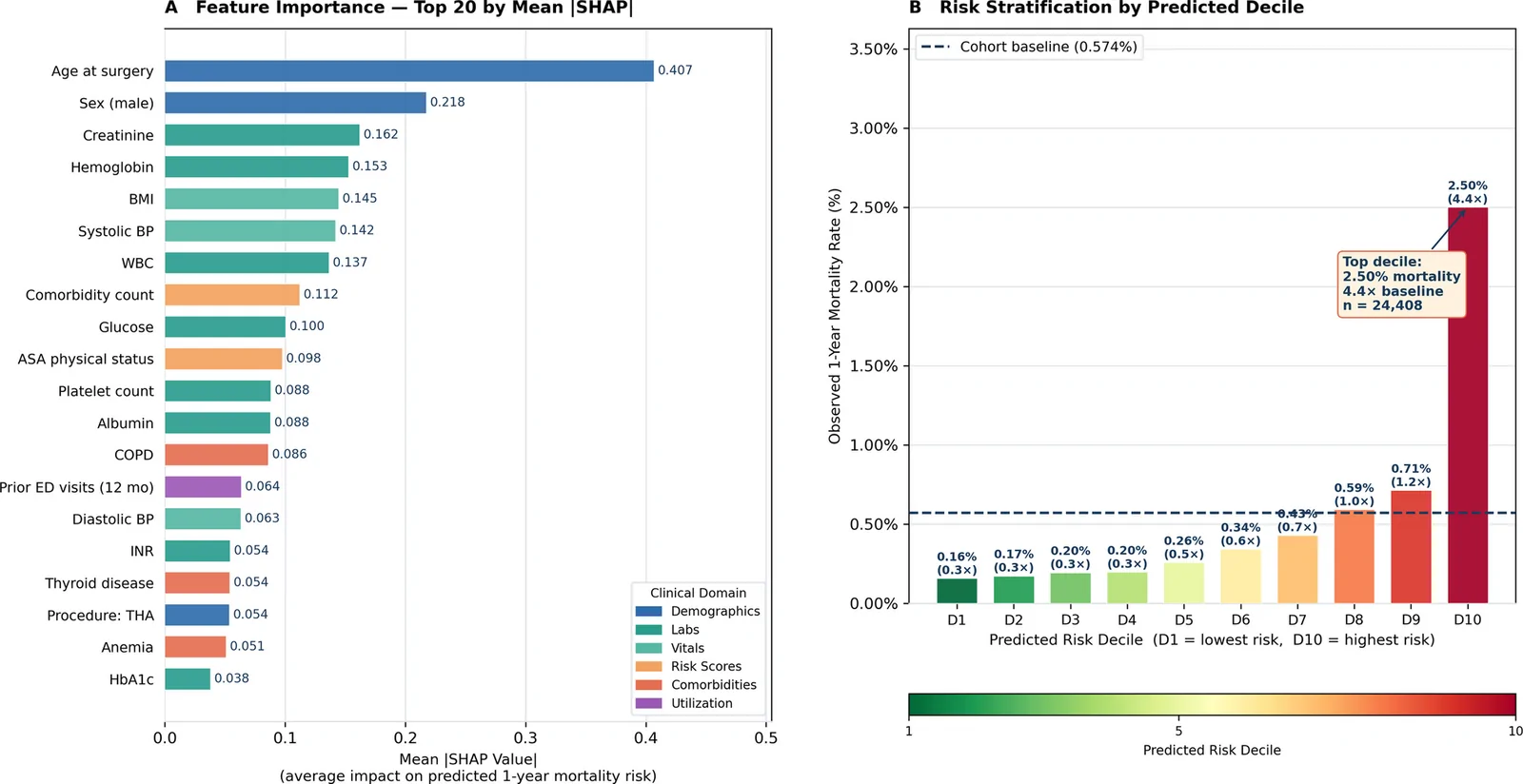
Feature importance and decile-level risk stratification for the XGBoost 1-year mortality prediction model. **A** Top 20 preoperative predictors ranked by mean absolute SHAP value, representing the average contribution of each feature to individual model predictions. Bars are color-coded by clinical domain: demographics (blue), laboratory values (teal), vital signs (green), risk scores/composite indices (yellow), comorbidities (orange), healthcare utilization (purple), and missing value indicators (gray). **B** Observed 1-year all-cause mortality rate stratified by predicted risk decile (D1 = lowest risk, D10 = highest risk), where deciles are defined by predicted-probability bins. Fold enrichment relative to the cohort baseline rate (0.574%, horizontal dashed line) is displayed in parentheses above each bar. Bar color transitions from green (lowest risk) to red (highest risk). The top decile (*n* = 24,408) had an observed mortality rate of 2.50% (4.4-fold enrichment)

Observed 1-year mortality by predicted risk quintile is shown in Fig. 1B. Patients in the lowest three quintiles (P0–P60) had observed mortality rates of 0.169%, 0.198%, and 0.298%, corresponding to 0.3 ×, 0.3 ×, and 0.5 × the cohort baseline, respectively. The fourth quintile (P60–P80) had an observed rate of 0.485% (0.8 × baseline). The highest quintile (P80–P100, *n* = 56,432) had an observed rate of 1.488% (2.6 × baseline). At the decile level (Fig. 2B), the highest-risk decile had an observed mortality of 2.50% (4.4-fold enrichment), and the highest-risk 5th percentile had an observed mortality of 3.57% (6.2-fold enrichment). The top 15 predictors by mean absolute SHAP value are shown in Fig. 2A. Age at surgery was the dominant predictor (mean |SHAP| 0.407), followed by male sex (0.218), creatinine (0.162), hemoglobin (0.153), BMI (0.145), systolic blood pressure (0.142), and WBC (0.137). Together, laboratory values accounted for approximately 28% of total model predictive weight, and demographic features (age, sex, procedure type) accounted for 24%. Comorbidity count (mean |SHAP| 0.112) outperformed all individual comorbidity flags, including the highest-ranked individual comorbidity (COPD; mean |SHAP| 0.086). Prior emergency department visits ranked 14th overall (mean |SHAP| 0.064).

In a post hoc sensitivity analysis, SHAP values were computed separately for the THA and TKA patient subsets to assess whether a single combined model was appropriate across procedure types. The top 10 predictors were identical in rank order across both procedure types (Supplemental Fig. S2). The only divergence exceeding 3 rank positions among the top 20 predictors was thyroid disease (rank 15 in TKA versus rank 19 in THA), a lower-weighted predictor contributing minimal predictive weight in either subgroup. Subgroup AUROCs were 0.778 for THA and 0.746 for TKA, confirming consistent discrimination across procedure types and supporting the combined modeling approach.

### Decision curve analysis

**Fig. 3:**
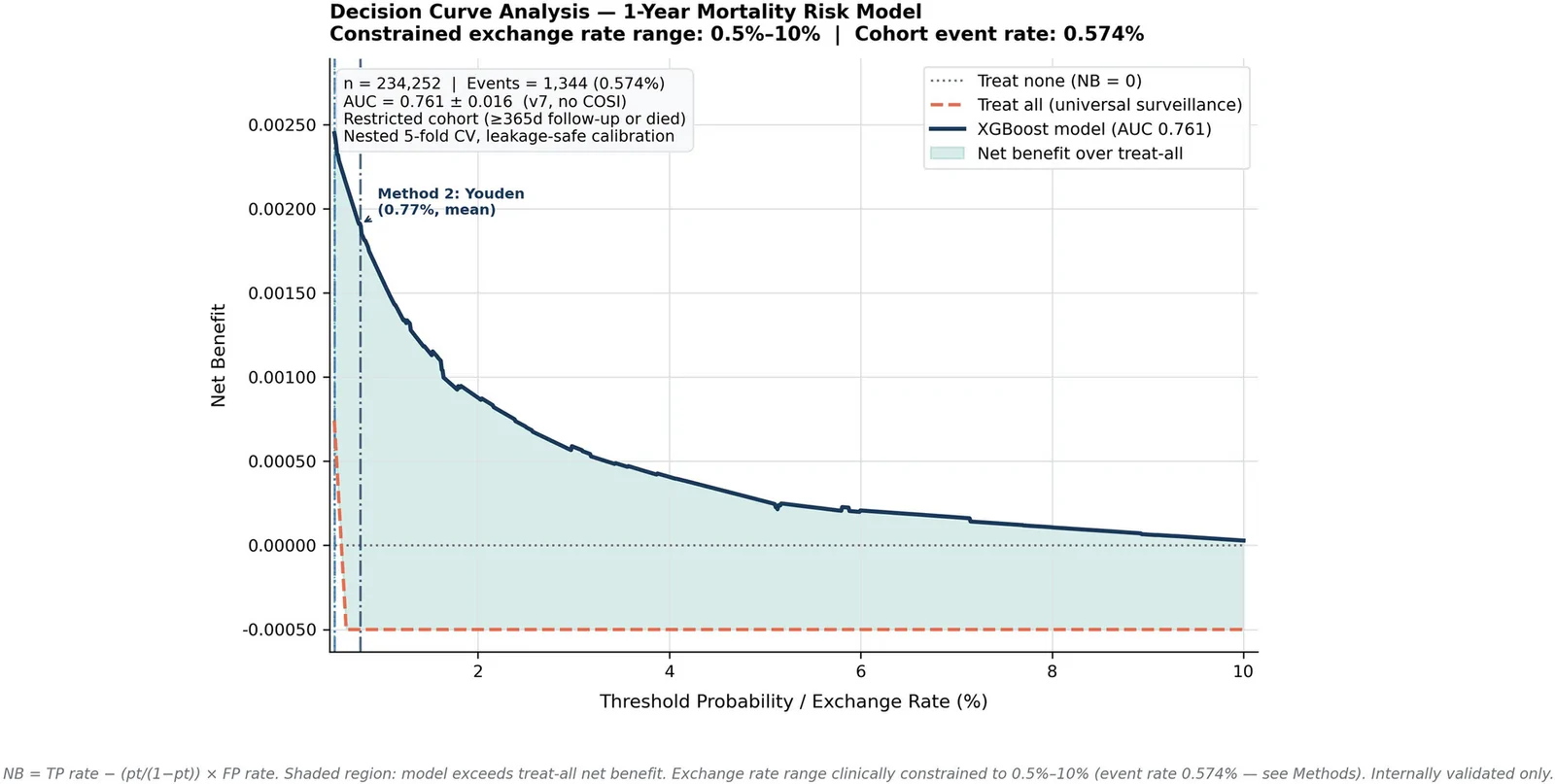
Decision curve analysis for the XGBoost 1-year mortality prediction model. Net benefit is plotted as a function of threshold probability across the pre-specified clinically actionable risk range (0.5%–10.0%). The solid curve represents the XGBoost model; the gray dashed line represents the treat-all strategy; the horizontal line at zero represents the treat-none strategy. Vertical reference lines indicate the DCA net benefit-maximizing threshold (0.500%) and the cross-validated Youden index threshold (0.767%)

Decision curve analysis is shown in Fig. 3. The XGBoost model demonstrated a positive net benefit that exceeded both the treat-all and treat-none strategies across the entire prespecified risk range of 0.5% to 10.0%. At a risk threshold of 0.500%, the model’s net benefit was 0.00245, compared with 0.00074 for the treat-all strategy (absolute advantage + 0.00171). At the Youden threshold (0.767%), the model’s net benefit was 0.00191, compared with − 0.00194 for the treat-all strategy (absolute advantage + 0.00385). At a risk threshold of 10.0%, the treat-all net benefit was − 0.10474, while the model retained a net benefit of 0.00003. The treat-none strategy yielded a net benefit of 0.000 across the full range.

### Threshold derivation

Three complementary thresholds were derived from cross-validated out-of-fold predictions. The DCA net-benefit-maximizing threshold of 0.500% identified 70,412 patients (30.0%) as elevated risk (sensitivity 68.7%, specificity 70.2%) and showed net benefit exceeding the treat-all strategy across the pre-specified risk range. The cross-validated Youden index threshold of 0.767% (SD 0.275% across folds) flagged 38,145 patients (16.3%), with sensitivity 54.8% and specificity 83.9%. The sensitivity-anchored threshold of 0.235% achieved 90.1% sensitivity at 33.2% specificity, identifying 156,813 patients (66.9%) for enhanced surveillance.

### Partial dependence analysis

**Fig. 4:**
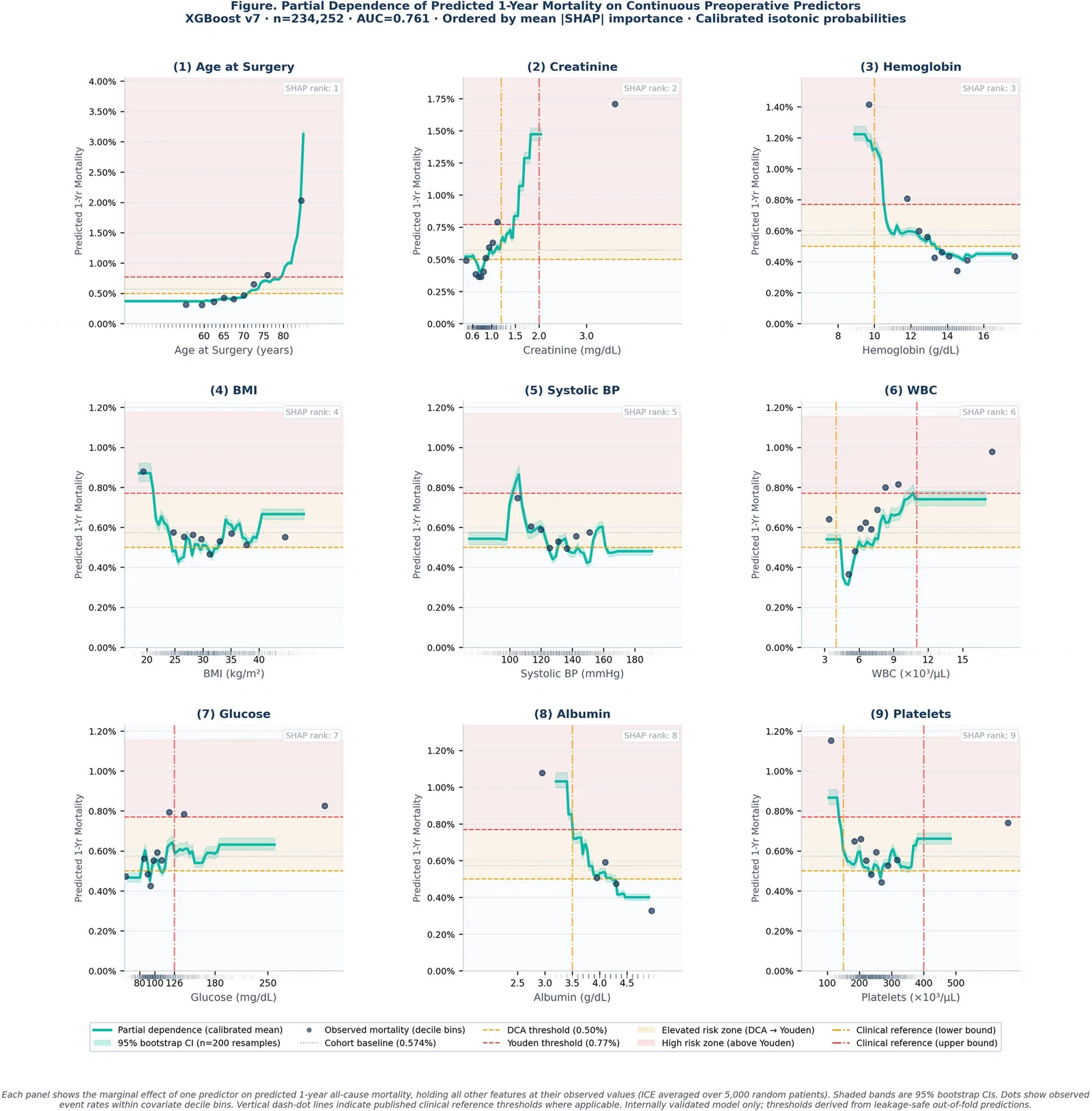
Partial dependence plots for the nine highest-ranked continuous predictors. Each panel displays the marginal effect of a single predictor on predicted 1-year mortality, averaged across all other features in the model. Horizontal dashed lines indicate the DCA net benefit-maximizing threshold (0.500%, blue) and the cross-validated Youden index threshold (0.767%, red). Rug plots along the x-axis show the distribution of observed values. Predictors are ordered by mean absolute SHAP value: (1) age at surgery, (2) creatinine, (3) hemoglobin, (4) BMI, (5) systolic blood pressure, (6) WBC, (7) glucose, (8) albumin, (9) platelet count

Partial dependence plots for the nine highest-ranked continuous predictors are shown in Fig. 4. Age at surgery showed the steepest marginal effect: predicted 1-year mortality was 0.374% at age 60, 0.455% at age 70, 0.685% at age 75, 0.930% at age 80, and 1.908% at age 85, crossing the DCA net-benefit threshold (0.500%) at 71.1 years and the Youden threshold (0.767%) at 79.0 years. Creatinine showed a monotonic positive relationship, with predicted mortality of 0.453% at 0.7 mg/dL, 0.651% at 1.2 mg/dL, 0.833% at 1.5 mg/dL, and 1.473% at 2.0 mg/dL, crossing the Youden threshold at 1.5 mg/dL. Hemoglobin showed an inverse relationship, with predicted mortality of 1.223% at or below 9.0 g/dL, 1.131% at 10.0 g/dL, 0.582% at 11.0 g/dL, and 0.450% at 14.0 g/dL, crossing the Youden threshold at 10.6 g/dL. Albumin also showed an inverse relationship: predicted mortality was 1.032% at or below 3.0 g/dL, 0.852% at 3.5 g/dL, and 0.518% at 4.0 g/dL, crossing the Youden threshold at 3.5 g/dL. BMI exhibited a U-shaped pattern, with peak predicted mortality of 0.871% at or below BMI 20 kg/m^2^ and a nadir of 0.428% at BMI 25.9 kg/m^2^; the mid-to-upper range (BMI 30–45) remained between 0.514% and 0.666%. Systolic blood pressure showed a bidirectional pattern, with peak predicted mortality of 0.865% at 105 mmHg and a nadir of 0.422% at 150 mmHg; the effect remained below the Youden threshold throughout the observed distribution. WBC showed an overall range of 0.314% to 0.773% across the cohort distribution, with the Youden threshold reached at the upper bound of the observed range (10.5 × 10^3^/µL). Glucose contributed a modest, attenuated effect, ranging from 0.444% to 0.648% across the observed distribution, without crossing the Youden threshold at any point. Platelet count showed elevated predicted mortality (0.866%) at counts at or below 119 × 10^3^/µL, declining to 0.601% in the normal range and rising modestly to 0.661% above 400 × 10^3^/µL.

## Discussion

The principal contribution of this study is not only that 1-year mortality after primary THA and TKA can be predicted with good discrimination (AUROC 0.761), representing a + 0.113 AUC improvement over comorbidity count alone and a + 0.130 AUC improvement over an ICD-10-based ASA physical status proxy, but also that the model identifies a coherent preoperative profile of patients at highest risk of mortality. Age was the dominant predictor, but higher predicted mortality was also independently associated with impaired renal function, anemia, hypoalbuminemia, and greater recent healthcare utilization, each of which has been identified as a predictor of adverse outcomes after TJA in prior literature and collectively reflects the physiologic reserve framework underlying prior personalized mortality models in this population [11–15]. In practical terms, older patients whose profiles also include creatinine ≥ 1.5 mg/dL, hemoglobin ≤ 10.6 g/dL, or albumin ≤ 3.5 g/dL constitute a subgroup for whom proceeding directly to elective arthroplasty would be difficult to justify without further preoperative optimization. These values should be interpreted as model-derived markers of limited reserve rather than hard contraindications.

This becomes particularly relevant in the current reimbursement environment, where hospitals increasingly bear episode-level accountability for outcomes and spending after joint replacement. Arthroplasty has long been among Medicare’s most common and costly procedures, and the Centers for Medicare & Medicaid Services (CMS) has progressively expanded episode-based payment models for these cases, from the Bundled Payments for Care Improvement initiative to the Comprehensive Care for Joint Replacement (CJR) model and now the Transforming Episode Accountability Model (TEAM) [1, 16–18]. TEAM is now a mandatory five-year model running from January 1, 2026, through December 31, 2030, for selected acute care hospitals. It includes episodes of lower-extremity joint replacement and uses risk-adjusted target pricing based on beneficiary- and hospital-level factors. Within this policy framework, postoperative complications, utilization, and recovery trajectories carry consequences not only for patients but also for hospital financial performance. For that reason, preoperative mortality stratification has relevance beyond bedside counseling. If risk is assessed crudely, bundled-payment incentives may encourage hospitals to associate older age or medical complexity with poor surgical candidacy among individuals who may derive substantial benefits from arthroplasty. In contrast, a more individualized estimate of mortality risk may better inform decisions about medical optimization, surgical timing, and alternative treatment strategies, while reducing the likelihood that bundled-payment incentives are addressed through indiscriminate patient selection [19, 20]. Framed this way, the policy value of preoperative risk estimation is not simply that it predicts adverse outcomes, but that it may help health systems pursue value-based care that is more clinically grounded and potentially more equitable.

Accordingly, the most appropriate use of this model is not a blanket denial of TJA to older or medically complex patients. The key clinical value is distinguishing patients with potentially reversible contributors to risk, particularly anemia, poor nutritional status, and unresolved renal dysfunction, from those whose risk reflects more global frailty and limited physiologic reserve. The former group may become reasonable surgical candidates after optimization. The latter group may warrant delaying or deferring the case because the surgical burden may outweigh the likelihood of meaningful long-term benefit. Additionally, the choice of a 1-year mortality endpoint was deliberate. Current CMS episode windows are shorter, but a patient who dies within the first postoperative year is unlikely to achieve the durable pain relief and functional improvement that justify elective arthroplasty. For that reason, 1-year mortality should be viewed as complementary to, rather than misaligned with, current reimbursement metrics.

Deploying the work as a freely accessible, real-time calculator increases its translational relevance [21]. Many arthroplasty machine-learning studies stop at retrospective performance benchmarks, whereas a tool built from routinely available EMR data can be used for case selection, optimization, resource allocation, and discharge planning [22]. This may be especially important for community, rural, and safety-net hospitals participating in mandatory bundled-payment models without extensive in-house analytic infrastructure. The three derived threshold-based operating points are intended to guide rather than govern clinical decision-making. In practical terms, a predicted probability below 0.235% identifies patients at low risk, above 0.767% identifies those at highest risk warranting close preoperative scrutiny, and the intermediate range flags patients who may benefit from targeted optimization. What matters clinically is the broader risk group a patient occupies rather than the precise probability value. Observed 1-year mortality in the highest predicted decile was 2.50%, representing 4.4-fold enrichment over the cohort baseline, a difference that is more meaningful than the distinction between any two adjacent probability values.

This work exists within a broader trajectory of machine learning adoption across orthopaedic care. Recent advances have demonstrated the utility of deep learning for automated classification of knee osteoarthritis severity and detection of metabolic bone disease from plain radiographs [23, 24]. Preoperative outcome prediction represents a complementary but distinct application, shifting the analytical focus from retrospective diagnostic classification to prospective patient-level risk stratification at the point of care. Where imaging-based models identify disease that is already present, preoperative prediction models aim to quantify the risk of outcomes that have not yet occurred, informing decisions that can still be changed, an approach the present work extends to one of the most consequential preoperative questions in elective joint replacement surgery.

This study has several strengths, including the use of a large, multi-institutional dataset that provides broad representation across practice settings and patient populations. The nested cross-validation framework ensures that both model predictions and calibration are evaluated on held-out data, minimizing optimism bias. The explicit restriction to patients with verifiable follow-up addresses a key limitation of EMR-based mortality studies. Finally, integrating model development with real-time deployment enhances the translational relevance of the work. Several limitations should be considered. First, while the model has been internally validated on a large, geographically diverse multi-institutional cohort using a nested cross-validation framework, it has not been externally validated in an independent dataset. Derivation from a multi-institutional network provides greater heterogeneity than single-institution studies. However, all participating institutions share the TriNetX data infrastructure and coding conventions, and performance in datasets derived from claims databases, independent EHR systems, or non-US healthcare settings cannot be assumed. External validation in independent cohorts remains a prerequisite before clinical adoption, and the publicly deployed web-based calculator is intended to facilitate prospective validation efforts. Second, TriNetX data are subject to coding variability and incomplete capture of care outside participating institutions, and mortality dates are limited to month and year due to de-identification. Similarly, cohort identification relied on ICD-10-PCS procedure codes rather than CPT codes, which may have resulted in incomplete capture of qualifying procedures at institutions with incomplete ICD-10-PCS documentation. The study period spans 2000 to 2025, during which surgical technique, perioperative protocols, and institutional mortality rates for TJA have evolved substantially. Although 88.9% of procedures were performed in 2015 or later under standardized ICD-10-PCS coding practices, model performance in the pre-2015 subgroup may differ from contemporary cases. Third, while restricting the cohort to patients with verifiable 1-year survival status avoids outcome misclassification inherent to unverified administrative data, patients with fragmented care or lower healthcare engagement are more likely to be excluded, and the observed mortality rate of 0.574% is likely conservative relative to the broader TJA population. Fourth, while formal frailty indices and patient-reported functional status were not included, the combination of age, aggregate comorbidity burden, continuous laboratory markers of organ function and nutritional status, and prior healthcare utilization was designed to approximate the construct of physiologic reserve that frailty instruments measure. Socioeconomic variables are unavailable in the TriNetX de-identified export, and formal frailty instruments require administration of items not routinely captured in structured EHR fields, both of which would preclude real-time deployment in the current architecture. Fifth, while the AUROC of 0.761 reflects meaningful discrimination, it also reflects the difficulty of predicting rare events in an elective surgical population. Finally, the focus on a 1-year endpoint does not directly address shorter-term outcomes aligned with current payment models, which warrant separate evaluation.

A logical next step is prospective clinical testing to evaluate whether integrating this risk estimate into preoperative pathways changes provider behavior and patient outcomes. The deployed calculator was designed to facilitate this directly, and future studies should assess whether its use alters rates of preoperative optimization, case delays or deferrals, post-acute utilization, episode spending, and access disparities under contemporary bundled-payment models. Ultimately, the goal is to ensure that risk stratification tools support more informed surgical decision-making rather than serving as a barrier to care for older or medically complex patients who stand to benefit from arthroplasty.

## Conclusion

This study shows that 1-year mortality after primary THA and TKA can be estimated with reasonable accuracy from routinely available preoperative data, with risk largely driven by age and physiologic status rather than isolated comorbidities. By translating these factors into individualized risk estimates, the model provides a framework for aligning surgical decision-making, preoperative optimization, and perioperative resource use with a patient’s underlying risk profile. For most patients, this supports targeted optimization and planning. However, for a smaller subset at the highest risk, it may also prompt reconsideration of surgical timing or alternative management strategies. As value-based care models continue to evolve, tools that quantify patient-specific risk at the point of care may play an important role in improving both outcomes and the appropriateness of surgical intervention.

## Supplementary Information

Supplementary Material 1.

## Data Availability

The datasets analyzed were obtained from the TriNetX Research Network (Cambridge, MA, USA), a federated platform aggregating de-identified electronic health records from U.S. healthcare organizations. Restrictions apply to the use of these data as they are supplied by TriNetX under institutional data use agreements and are not publicly available. Aggregated, de-identified data may be available from the corresponding author upon reasonable request and with the permission of TriNetX.

## References

[1] Sloan M, Premkumar A, Sheth NP. Projected volume of primary total joint arthroplasty in the U.S., 2014 to 2030. J Bone Joint Surg Am. 2014;100(17):1455. 10.2106/JBJS.17.01617. 30180053

[2] Pan X, Turan O, Rullan PJ, Simmons H, Emara AK, Piuzzi NS. 30-Days to 10-Years Mortality Rates following Total Knee Arthroplasty: A Systematic Review and Meta-Analysis of the Last Decade (2011–2021). J Knee Surg. 2023;36(13):1323–40. 10.1055/a-1911-3892. (Epub 2022 Jul 28 PMID: 35901803). 35901803

[3] Jämsen E, Puolakka T, Eskelinen A, Jäntti P, Kalliovalkama J, Nieminen J, et al. Predictors of mortality following primary hip and knee replacement in the aged. A single-center analysis of 1,998 primary hip and knee replacements for primary osteoarthritis. Acta Orthop. 2013;84(1):44–53. 10.3109/17453674.2012.752691. 23244785 PMC3584602

[4] McConaghy KM, Orr MN, Emara AK, Sinclair ST, Klika AK, Piuzzi NS. Can extant comorbidity indices identify patients who experience poor outcomes following total joint arthroplasty? Arch Orthop Trauma Surg. 2023;143(3):1253–63. 10.1007/s00402-021-04250-y. 34787694

[5] Kerr MM, Graves SE, Duszynski KM, Inacio MC, de Steiger RN, Harris IA, et al. Does a Prescription-based Comorbidity Index Correlate with the American Society of Anesthesiologists Physical Status Score and Mortality After Joint Arthroplasty? A Registry Study. Clin Orthop Relat Res. 2021;479(10):2181–90. 10.1097/CORR.0000000000001895. (PubMedPMID:34232146;PubMedCentralPMCID:PMC8445560). 34232146 PMC8445560

[6] Buddhiraju A, Shimizu MR, Chen TLW, Seo HH, Bacevich BM, Xiao P, et al. Comparing prediction accuracy for 30-day readmission following primary total knee arthroplasty: the ACS-NSQIP risk calculator versus a novel artificial neural network model. Knee Surg & Relat Res. 2025;37(1):3. 10.1186/s43019-024-00256-z. 39806502 PMC11727824

[7] Gimm G, Jeon B, Kim SE, Kim BS, Han HS, Kim S. A development of machine learning models to preoperatively predict insufficient clinical improvement after total knee arthroplasty. J Orthop Surg Res. 2025;20(1):778. 10.1186/s13018-025-06206-z. 40835961 PMC12366143

[8] Zampogna B, Torre G, Zampoli A, Parisi F, Ferrini A, Shanmugasundaram S, et al. Can machine learning predict the accuracy of preoperative planning for total hip arthroplasty, basing on patient-related factors? An explorative investigation on supervised machine learning classification models. J Clin Orthop Trauma. 2024;53:102470. 10.1016/j.jcot.2024.102470. 39045495 PMC11261062

[9] Abraham VM, Booth G, Geiger P, Balazs GC, Goldman A. Machine-learning Models Predict 30-Day Mortality, Cardiovascular Complications, and Respiratory Complications After Aseptic Revision Total Joint Arthroplasty. Clin Orthop Relat Res. 2022;480(11):2137–45. 10.1097/CORR.0000000000002276. (PubMedPMID:35767804;PubMedCentralPMCID:PMC9555902). 35767804 PMC9555902

[10] Karlin EA, Lin CC, Meftah M, Slover JD, Schwarzkopf R. The impact of machine learning on total joint arthroplasty patient outcomes: a systemic review. J Arthroplasty. 2023;38(10):2085–95. 10.1016/j.arth.2022.10.039. 36441039

[11] Trela-Larsen L, Kroken G, Bartz-Johannessen C, Sayers A, Aram P, McCloskey E, et al. Personalized estimation of one-year mortality risk after elective hip or knee arthroplasty for osteoarthritis: JointCalc model development and validation using the National Joint Registry and the Norwegian Arthroplasty Register. Bone & Joint Research. 2020;9(11):808–20. 10.1302/2046-3758.911.BJR-2020-0343.R1. 33179531 PMC7672327

[12] McIsaac DI, Beaulé PE, Bryson GL, Walraven CV. The impact of frailty on outcomes and healthcare resource usage after total joint arthroplasty: a population-based cohort study. Bone Joint J. 2016;98-B(6):799–805. 10.1302/0301-620X.98B6.37124. 27235523

[13] Cavanaugh PK, Chen AF, Rasouli MR, Post ZD, Orozco FR, Ong AC. Complications and mortality in chronic renal failure patients undergoing total joint arthroplasty: a comparison between dialysis and renal transplant patients. J Arthroplasty. 2016;31(2):465–72. 10.1016/j.arth.2015.09.003. 26454568

[14] Zhang FQ, Yang YZ, Li PF, Ma GR, Zhang AR, Zhang H, et al. Impact of preoperative anemia on patients undergoing total joint replacement of lower extremity: a systematic review and meta-analysis. J Orthop Surg Res. 2024;19(1):249. 10.1186/s13018-024-04706-y. 38637795 PMC11027536

[15] Fryhofer GW, Sloan M, Sheth NP. Hypoalbuminemia remains an independent predictor of complications following total joint arthroplasty. J Orthop. 2019;16(6):552–8. 10.1016/j.jor.2019.04.019. 31660022 PMC6806651

[16] Comprehensive Care for Joint Replacement Model | CMS. Available from: https://www.cms.gov/priorities/innovation/innovation-models/cjr. Cited 2026 Apr 10.

[17] Bundled Payments for Care Improvement Initiative (BPCI) Fact Sheet | CMS. Available from: https://www.cms.gov/newsroom/fact-sheets/bundled-payments-care-improvement-initiative-bpci-fact-sheet. Cited 2026 Apr 10.

[18] TEAM (Transforming Episode Accountability Model) | CMS. Available from: https://www.cms.gov/priorities/innovation/innovation-models/team-model. Cited 2026 Apr 10.

[19] Wilcock AD, Barnett ML, McWilliams JM, Grabowski DC, Mehrotra A. Hospital responses to incentives in episode-based payment for joint surgery: a controlled population-based study. JAMA Intern Med. 2021;181(7):932–40. 10.1001/jamainternmed.2021.1897. 33999159 PMC8129900

[20] Thirukumaran CP, Glance LG, Cai X, Kim Y, Li Y. Penalties and rewards for safety net vs non–safety net hospitals in the first 2 years of the comprehensive care for joint replacement model. JAMA. 2019;321(20):2027–30. 10.1001/jama.2019.5118. 31135838 PMC6547113

[21] Zotov E, Hills AF, de Mello FL, Aram P, Sayers A, Blom AW, et al. JointCalc: a web-based personalised patient decision support tool for joint replacement. Int J Med Inform. 2020;142:104217. 10.1016/j.ijmedinf.2020.104217. 32853974 PMC7607377

[22] Dijkstra H, van de Kuit A, de Groot TM, Canta O, Groot OQ, Oosterhoff JH, et al. Systematic review of machine-learning models in orthopaedic trauma: an overview and quality assessment of 45 studies. Bone & Joint Open. 2024;5(1):9–19. 10.1302/2633-1462.51.BJO-2023-0095.R1. 38226447 PMC10790183

[23] Naguib SM, Saleh MK, Hamza HM, Hosny KM, Kassem MA. A new superfluity deep learning model for detecting knee osteoporosis and osteopenia in X-ray images. Sci Rep. 2024;14(1):25434. 10.1038/s41598-024-75549-0. 39455632 PMC11511848

[24] Naguib SM, Kassem MA, Hamza HM, Fouda MM, Saleh MK, Hosny KM. Automated system for classifying uni-bicompartmental knee osteoarthritis by using redefined residual learning with convolutional neural network. Heliyon. 2024;10(10):e31017. 38803931 10.1016/j.heliyon.2024.e31017 PMC11128872

