# Supplementary material for: Advancing preoperative planning technology in total joint arthroplasty with a real-time machine learning calculator: 1-year mortality risk in a value-based care era
Source: Arthroplasty. 2026 Sep 9;8:70. doi: 10.1186/s42836-026-00428-0 (PMC13555866; doi:10.1186/s42836-026-00428-0)
Supplement: Supplementary file 1 [file 42836_2026_428_MOESM1_ESM.docx]

**Supplementary material**

**Fig. S1. Receiver operating characteristic curve.** Receiver operating characteristic (ROC) curves for the XGBoost model and two baseline comparators, ICD-10-based ASA physical status proxy and comorbidity count alone, derived from leakage-safe nested 5-fold cross-validated out-of-fold predictions (*n* = 234,252). The shaded band represents ±1 SD of fold-level AUC across the five outer folds (range 0.744–0.787; standard deviation [SD]: 0.016) and reflects variability across folds rather than a formal confidence interval. Threshold markers indicate the operating points corresponding to the three derived decision thresholds: the DCA net benefit-maximizing threshold (0.500%), the cross-validated Youden index threshold (0.767%), and the ≥90% sensitivity anchor (0.235%). AUC = area under the receiver operating characteristic curve; DCA = decision curve analysis; SHAP = SHapley Additive exPlanations; THA = total hip arthroplasty; TKA = total knee arthroplasty.

**Fig. S2. SHAP feature importance by procedure type.** Top 15 preoperative predictors ranked by mean absolute SHAP value, computed separately for the full cohort (*n* = 234,252), total hip arthroplasty (THA; *n* = 94,048), and total knee arthroplasty (TKA; *n* = 140,204) subgroups using the TreeExplainer algorithm applied to a random sample of 5,000 patients per subgroup. Rankings were highly consistent across procedure types; the only divergence exceeding 3 rank positions among the top 20 predictors was thyroid disease (rank 15 in TKA versus rank 19 in THA). SHAP = SHapley Additive exPlanations.
